# Highlights on Steroidal Arylidene Derivatives as a Source of Pharmacologically Active Compounds: A Review

**DOI:** 10.3390/molecules26072032

**Published:** 2021-04-02

**Authors:** Vanessa Brito, Gilberto Alves, Paulo Almeida, Samuel Silvestre

**Affiliations:** 1 CICS-UBI—Health Sciences Research Centre, University of Beira Interior, Av. Infante D. Henrique, 6200-506 Covilhã, Portugal; vanessa_12_479@hotmail.com (V.B.); gilberto@fcsaude.ubi.pt (G.A.); 2 CNC—Center for Neuroscience and Cell Biology, University of Coimbra, 3004-517 Coimbra, Portugal

**Keywords:** steroids, arylidenesteroids, aldol condensation, bioactivity, heterocycles

## Abstract

Steroids constitute a unique class of chemical compounds, playing an important role in physiopathological processes, and have high pharmacological interest. Additionally, steroids have been associated with a relatively low toxicity and high bioavailability. Nowadays, multiple steroidal derivatives are clinically available for the treatment of numerous diseases. Moreover, different structural modifications on their skeleton have been explored, aiming to develop compounds with new and improved pharmacological properties. Thus, steroidal arylidene derivatives emerged as a relevant example of these modifications. This family of compounds has been mainly described as 17β-hydroxysteroid dehydrogenase type 1 and aromatase inhibitors, as well as neuroprotective and anticancer agents. Besides, due to their straightforward preparation and intrinsic chemical reactivity, steroidal arylidene derivatives are important synthetic intermediates for the preparation of other compounds, particularly bearing heterocyclic systems. In fact, starting from arylidenesteroids, it was possible to develop bioactive steroidal pyrazolines, pyrazoles, pyrimidines, pyridines, spiro-pyrrolidines, amongst others. Most of these products have also been studied as anti-inflammatory and anticancer agents, as well as 5α-reductase and aromatase inhibitors. This work aims to provide a comprehensive overview of steroidal arylidene derivatives described in the literature, highlighting their bioactivities and importance as synthetic intermediates for other pharmacologically active compounds.

## 1. Introduction

Steroids are natural products that share a 17-carbon-atom skeleton and are composed of four fused rings: three cyclohexanes (A, B, and C rings) and one cyclopentane (D ring). These compounds vary on the attached functional groups, their position, and configuration [1]. In addition, steroids represent a unique class of chemical products, playing an important role in several biological processes, being the most important group of regulatory and signaling molecules [2,3]. In fact, this class of compounds is widely found in animal and plant kingdoms. In humans, the different steroids are biosynthesized from cholesterol via several enzyme-mediated transformations. Usually, steroids are lipophilic and readily enter cells, being able to interact with nuclear receptors as well as with membrane proteins. Therefore, they are associated with most physiological functions and pathological conditions. In addition, due to their low toxicity, less vulnerability to multidrug resistance, and high bioavailability [4,5,6,7,8], steroid-based therapeutic drugs have called attention to the scientific academia and industry for a long time. Consequently, steroidal derivatives constitute an important class of pharmaceutically active compounds for a large number of diseases, including brain tumors, breast and prostate cancers, osteoarthritis, adrenal insufficiencies, fungal and microbial infections, in addition to cardiovascular and autoimmune diseases [3,9,10,11,12,13].

Due to their relevance, several modified steroids have been synthesized and biologically evaluated, being verified that their relevant pharmacological properties depend on the structural features of the steroidal four-ring skeleton and side-chain [5,14]. In fact, even a minor structural variation on the steroidal nucleus can lead to marked changes in their physiological activity [15]. Therefore, aiming to improve their pharmacological properties and/or develop compounds with different bioactivities, structural modifications of steroids have been an important focus of research over the last decades [16,17,18,19,20]. 

One of the diverse groups of modified steroids is arylidenesteroids, which are the focus of the present review. Generally, arylidene derivatives have been associated with a wide range of pharmacological activities, including antifungal, antimicrobial, antioxidant, anti-arrhythmic, anticancer, antimalarial, and anti-inflammatory [21,22,23,24,25,26,27,28,29,30]. The general structure of these compounds comprises a natural or synthetic core bearing an aryl group linked through an exocyclic double bound, which is part of a α,β-unsaturated ketone system. An advantage of this system is the inherent chemical reactivity due to the simultaneous presence of electrophilic and nucleophilic points. Therefore, these derivatives are especially useful in the preparation of several bioactive heterocyclic systems [4,31,32]. Thus, considering the pharmacological relevance of steroids and the biological and synthetic value of arylidene derivatives, it is not surprising that several studies involved the synthesis and biological evaluation of steroidal arylidenes, despite some potential problems of these compounds. In fact, in addition to the known possible hormonal effects, usually, these steroids have higher lipophilicity and the consequent risk of bioavailability problems. Moreover, the presence and inherent reactivity of their α,β-unsaturated ketone system can also be associated with potential toxicity problems, including mutagenesis and carcinogenesis [31,32]. Despite this, over the years, various arylidenesteroids have been studied, namely as hydroxysteroid dehydrogenases, aromatase, and 5α-reductases inhibitors, as well as skeletal muscle relaxants, antimicrobial, neuroprotective, antiparasitic, and antiproliferative agents [17,33,34,35,36,37,38,39,40]. Additionally, other research works have been demonstrating their use as synthetic intermediates, mainly in the preparation of aza-heterocycles with relevant potential applications [41,42]. 

In the past few years, several reviews have been published reporting the importance and synthetic approaches for chemical modifications on the steroidal skeleton [43,44,45]. However, arylidene steroidal derivatives and their application were only superficially presented, being inexistent an extensive and comprehensive review on this important topic. Therefore, this review aims for the first time, to our knowledge, to highlight steroidal arylidene derivatives described in the literature, particularly 2- and 16*E*-arylideneandrostanes, 21*E*-arylidenepregnanes and 16*E*-arylidenoestranes, with relevant pharmacological activity, as well as their main chemical synthesis and application as synthetic intermediates of relevant bioactive molecules, particularly heterocyclic compounds.

## 2. Synthetic Approaches to Prepare Arylidene Steroidal Derivatives

Arylidenesteroids are usually obtained through an aldol condensation between a steroid and an aldehyde. In an aldol condensation, an enol or enolate reacts with a carbonyl in the presence of an acid or base catalyst to form a β-hydroxyaldehyde or a β-hydroxyketone, followed by dehydration to afford a conjugated enone [46]. More particularly, the reaction between an aldehyde/ketone capable of forming an enol/enolate and an aromatic carbonyl without an α-hydrogen (usually an aromatic aldehyde) is named Claisen–Schmidt condensation. In steroids, this reaction was mainly performed in C-16 or C-21 positions and less often in C-2.

**Figure 1:**
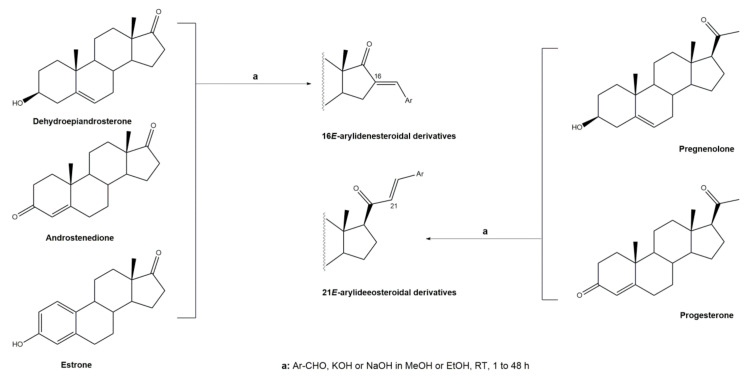
Claisen–Schmidt condensation to prepare 16*E*- and 21*E*-arylidenesteroids.

In general, the reactions to prepare arylidenesteroids occur at room temperature (RT), under basic catalysis, and the solvent is, in most cases, methanol (MeOH) or ethanol (EtOH) (Figure 1). The basicity of the reaction medium is conferred by sodium hydroxide (NaOH) or potassium hydroxide (KOH) [38,47,48,49,50,51] However, depending on reaction substrate and reagents, the duration of the reaction and observed yields are extremely variable. In fact, according to the literature, these reactions can take between 1 to 48 h to be concluded and yields ranging from 23 to 98% were described [32,42,52,53]. Androstane, pregnane, and estrane derivatives, namely dehydroepiandrosterone, androstenedione, estrone, pregnenolone, and progesterone have been described as the main starting materials.

Less often, the preparation of 2-arylidenesteroids from dehydroepiandrosterone (DHEA) derivatives, dihydrotestosterone (DHT), and cholestanone was described [31,54,55]. The synthesis of these derivatives was based on the classical approach for aldol condensation, occurring at RT and under basic catalysis, using MeOH or EtOH as solvent [31,54,55].

**Figure 2:**
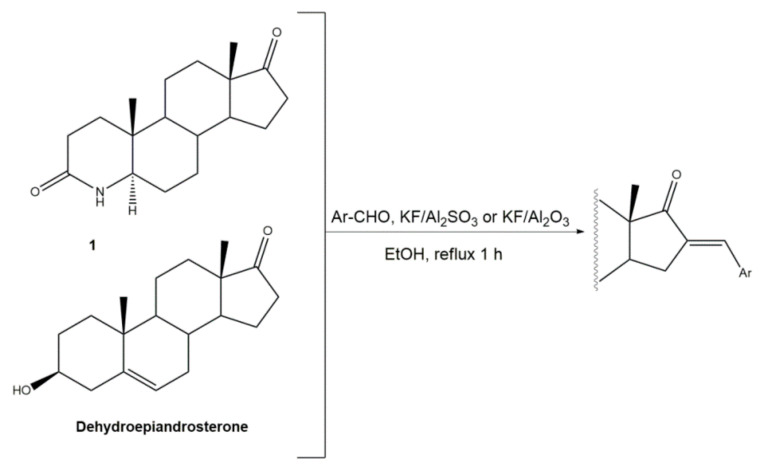
Aldol condensation using KF/Al_2_SO_3_ or KF/Al_2_O_3_ as catalysts in EtOH, under reflux, to synthesize 16*E*-arylidenesteroids from **1** and dehydroepiandrosterone.

**Figure 3:**
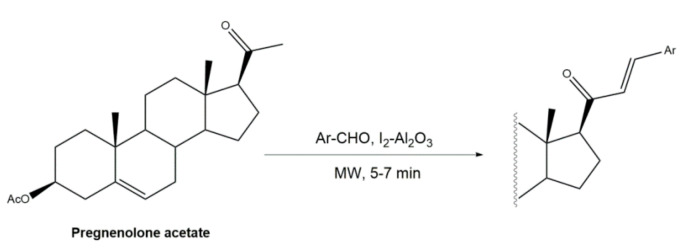
Preparation of 21*E*-arylidenepregnenolone acetate derivatives, catalyzed by I_2_-Al_2_O_3_ under microwave irradiation [52].

Interestingly, over the last years, other approaches different from the classical reaction conditions have been explored. As a representative example, Guo et al. performed the same reaction using MeOH as solvent and NaOH as a base, but at higher temperatures, to afford excellent yields (91–95%) [37]. In addition, Huang et al. and Yu et al. applied a different strategy to prepare the corresponding aldol condensation products from 4-azaandrost-3,17-dione (**1**) and dehydroepiandrosterone (DHEA) (Figure 2). As alternative for the use of NaOH or KOH, these authors used potassium fluoride on aluminum oxide (KF/Al_2_O_3_) as a recyclable basic catalyst and performed the reaction at EtOH under reflux temperature. Remarkably, shorter reaction times (1 h) were observed, and excellent yields of the products (90–94%) were achieved [41,56]. This transformation was also performed using molecular iodine-loaded aluminum oxide (I_2_-Al_2_O_3_) as heterogeneous catalyst and under microwave (MW) irradiation (Figure 3). These conditions allowed extremely short reaction times (5–7 min) and good yields of the products (79–85%) [52]. More recently, Mótyán et al. reported the synthesis of 16E-benzylidene-estrone-3-methyl ether derivatives by a MW-assisted (100 °C, 20 min) Claisen–Schmidt condensation, using EtOH as a solvent and KOH [53]. 

**Figure 4:**
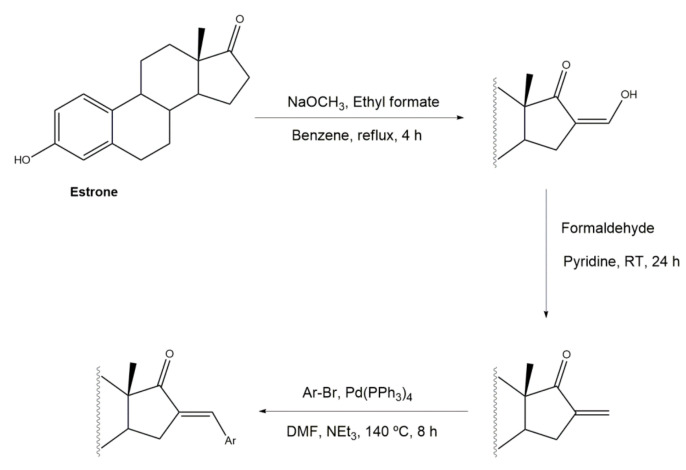
Alternative approach to obtain 16*E*-arylidenoestrane derivatives by the employment of Mizoroki–Heck reaction [54].

The most different approach to prepare steroidal arylidene derivatives was employed by Riebe et al., in the synthesis of 16*E*-arylidene-3-methoxy-estrone derivatives from commercially available 3-methoxyestrone (Figure 4). Firstly, an enol intermediate using sodium methoxide and ethyl formate in benzene and under reflux was prepared, followed by a reaction with formaldehyde in pyridine at room temperature. Then, a palladium-catalyzed Mizoroki–Heck reaction allowed the preparation of 16*E*-arylidenoestrane derivatives in fair to excellent yields (14–99%), strongly dependent on the electronic character of the aryl halide [54].

## 3. Bioactivity of 2- and 16*E*-arylideneandrostane Derivatives 

The 16*E*-arylideneandrostane steroidal skeleton has emerged as a relevant template to develop potential anticancer agents. Among all different types of arylidene steroids this is the most common. On the other hand, A-ring arylideneandrostane derivatives were also described but much less frequently [31,54,55]. In addition to cytotoxic effects, several studies reported other biological activities, such as aromatase inhibition, anti-inflammatory, neuroprotective and skeletal muscle relaxing activities and tissue-selective androgen receptor modulator effects [38,57,58]. A selection of the most relevant 16*E*-arylideneandrostane derivatives is presented in Table 1. The compounds selected presented better results than the respective positive controls in biological assays. When data for positive control is not present, the steroid with the best results is presented. Concerning relevant antiproliferative compounds, several arylideneandrostane derivatives, namely the steroids **2**–**7** (Table 1), have shown promising results [16,55,57,59,60,61]. In this context, Huang et al. reported the cytotoxic effects of a series of novel DHEA derivatives bearing a modified *v*-triazole ring at the C-16 position on different human cancer cells. Through the 3-(4,5-dimethylthiazol-2-yl)-2,5-diphenyltetrazolium bromide (MTT) colorimetric assay, these authors observed that compound **2** (Table 1), which contains a 4-iodophenyl system attached to the triazole ring, displayed the highest antiproliferative activity in HepG2 and MCF-7 cells [half maximal inhibitory concentration (IC_50_) of 9.10 and 9.18 μM, respectively]. Interestingly, these IC_50_ values are even lower than the estimated for the positive control 5-fluorouracil (5-FU), an antineoplastic drug widely used in clinical practice to treat multiple solid tumors [58]. Furthermore, flow cytometry experiments in the HepG2 cell line demonstrated that steroid **2** exerted antiproliferative effects by arresting cells in the G2 phase, inducing apoptosis [16]. The androstane derivative **3** (Table 1), bearing a 3-chlorobenzylidene at C-16, and analogues were synthesized by Vosooghi et al. and their cytotoxicity was evaluated through the MTT assay. The results evidenced that this steroid **3** was the most potent, especially against KB and T47-D cells (IC_50_ values of 0.6 and 1.7 μM, respectively) [60]. In another study, the amide androstane **4** with a chlorine atom attached to C-3 (Table 1) revealed potent antiproliferative effects against a panel of leukemia cell lines. In fact, the determined IC_50_ values for this steroid **4** against CCRF-CEM, K-562, RPMI-8226 and SR cells were 3.94, 2.61, 6.90, and 1.79 μM, respectively [61]. More recently, the synthesis and biological evaluation of ferrocenyl androstane conjugates was reported. The antiproliferative activity of these compounds was assessed and the steroid **5** (Table 1) was the most potent, being determined an IC_50_ of 1.2 μM in colon cancer HT-29 cells. This IC_50_ value is very relevant when compared with the positive control, cisplatin (Cis) (IC_50_ = 66 μM) [59]. Besides in vitro studies using cell lines, Chattopadhaya et al. also performed an in vivo cytotoxicity evaluation using a mouse model, the hollow fiber assay. In this study, interesting results were observed for the 3,4,5-trimetoxibenzylidene **6** (Table 1). In fact, for this steroid, a good score for inhibition of tumoral cells in the subcutaneous (S.C.) fiber implants (8/20) was determined, while for intraperitoneal (I.P.) fiber implants, the value significantly decreased (2/20) [57]. In a very similar study, Bansal et al. synthesized and tested a series of 16*E*-arylideneandrostene derivatives bearing tertiary amine functionalities. Of these, the aminosteroid **7** (Table 1), possessing a diethylaminoalkoxy group, was the most promising molecule with a score of 12 for I.P. and 8 for S.C. in the in vivo hollow fiber assay [62]. 

Several arylidenesteroids have been reported as aromatase inhibitors, particularly the 16*E*-arylideneandrostane derivatives **8**–**11** (Table 1). Aromatase is a cytochrome P450 enzyme (CYP19) responsible for the conversion of androgens into estrogens through an aromatization reaction. This enzyme plays a crucial role in endocrine physiology and in estrogen-dependent tumors such as breast and endometrial cancers [63]. Therefore, potent aromatase inhibitors (AIs) have been clinically relevant in the treatment of the referred diseases. However, the side effects of AIs used in clinical practice, such as muscle-skeletal pain, osteoporosis, and cardiovascular disease, are limiting and justify further research in this field [64,65,66,67,68]. In this context, the steroids **8**–**11** (Table 1) revealed potent aromatase inhibitory activity in in vitro assays. Noteworthy, the determined IC_50_ values for the enzyme inhibition are much lower than the observed for aminoglutethimide, used as a positive control [54,69,70,71].

16*E*-Arylidene steroidal derivatives were also studied as a new class of neuroprotective agents for the treatment of Alzheimer’s and Parkinson´s diseases. In this scope, Singh et al. reported some interesting results, being the 4-aza-16*E*-pyridylene derivative **12**, prepared from DHEA (Table 1), the most promising. This compound was found to be a relevant neuroprotective agent, producing effects on TNF-α levels (a pro-inflammatory cytokine) in brain serum of rats comparable to the standard drug celecoxib (CEL) and better results than dexamethasone (DEX) (Table 1) [72]. 

More recently, Li et al. prepared 2-arylideneandrostene derivatives and evaluated their potential anti-inflammatory properties, including their effect on NO release by microglia BV-2 cells activated by lipopolysaccharide (LPS), as well as their cytotoxicity. Among all synthesized derivatives, steroids **13** and **14** (Table 1), presenting, respectively, 3-chloro and 3,4,5-trimethoxy groups at C-2 position, displayed the most relevant results. In fact, the calculated IC_50_ values for anti-inflammatory effect were 2.69 and 3.28 μM, respectively, which were lower than the positive control, minocycline (5.97 μM) [73]. 

Despite these promising results, other studies carried out with 2- and 16*E*-arylideneandrostrane derivatives were described, but less relevant biological effects were observed [17,38,50,55,64,67]. In addition, Semenenko and coworkers prepared 16*E*-arylidenedehydroepiandrosterones, as well as 2-arylidenesteroidal derivatives of cholestanone, to be used as chiral dopants for cholesteric liquid crystal composition [74]. Another study reported the synthesis of a new series of quaternary ammonium salts of 16*E*-[4-(2-alkylaminoethoxy)-3-methoxybenzylidene]androstane derivatives as skeletal muscle relaxants. The biological results showed that these compounds produced varied degrees of muscle relaxant activity. However, they also inhibited acetylcholinesterase activity in low concentrations and therefore are not suitable for use as muscle relaxants [36]. Additionally, Acharya and coworkers prepared new 16*E*-arylidene steroids linked to nitrogen mustards as hybrid compounds and evaluated their cytotoxic effects aiming to develop antineoplastic agents against leukemia. However, the specificity of these compounds towards leukemia cells still remains unaffected and reduced potency was observed when compared to earlier reports for 16*E*-arylidene steroids [61,62]. Further studies suggested that these derivatives present different mechanisms of action in leukemia cells [17]. More recently, Brito et al. reported the synthesis, antiproliferative effects, and in silico studies of novel 16*E*-arylidene-4-azaandrost-5-enes as prostate cell growth inhibitors. Despite their less potent antiproliferative effects in comparison with the positive control (5-FU), an interesting selectivity toward cancer cell lines was found for all azaandrostenes, presenting low cytotoxicity in non-tumoral human fibroblasts. Furthermore, molecular docking studies predicted that these 4-azaandrostene derivatives can interact with 5β-reductase, a surrogate of 5AR, and with other common targets of steroidal drugs, which could be important for further studies [48].

## 4. Bioactivity of 21*E*-arylidenepregnene Derivatives

Arylidenesteroidal derivatives of progesterone and pregnenolone and other similar pregnanes constitute a smaller group than arylideneandrostanes, and they have mainly been studied as potential antitumoral agents. Of these, the most potent arylidenepregnene derivatives reported until now are presented in Table 2. The steroids **15** (with a 4-fluorophenyl group attached to C-21) and **16** (bearing a 4-nitrophenyl group attached to C-21 and an imine group at C-20) were prepared and tested by Banday and coworkers. Their effects on tumoral cell proliferation were assessed through National Cancer Institute-60 (NCI-60) Human Tumor Cell Lines Screen. This NCI-60 method involves 60 different human tumor cell lines, representing leukemia, melanoma, and cancers of the lung, colon, brain, ovary, breast, prostate, and kidney cancers, to identify and characterize novel compounds with growth inhibition or killing of tumor cells. This method was designed to screen up to 3000 small molecules for potential anticancer activity in a short period of time [75]. The best results for compounds **15** and **16** were observed in HCT-15 and MCF-7 cell lines, respectively, being determined an IC_50_ value of 0.81 μM for compound **15** in HCT-15 cells, and an IC_50_ value of 0.60 μM for the 21*E*-arylidenepregnene derivative **16** in MCF-7 cells [69]. A more recent study described the synthesis of novel 4′-acylamino modified 21*E*-benzylidene steroidal derivatives and their cytotoxic activity. The toxicity of these steroids was assessed by a brine shrimp microtiter-plate method and by the MTT assay against HeLa and MCF-7 cells [18,38]. Within these arylidenesteroids, the 21*E*-4-(2-fluorobenzamido)benzylidene derivative **17** (Table 2) seemed to be the most active. In fact, at a concentration of 30 μg·mL^−1^, this steroid originated a 58 and 64% growth inhibition of HeLa and MCF-7 cells, respectively, and their toxicity against brine shrimp was considered weak (median lethal concentration (LC_50_) > 50 μg·mL^−1^) [38]. Interestingly, Abood and coworkers reported the synthesis and antimicrobial study of 2,21*E*-arylidene progesterone derivatives. These compounds were tested using *Streptococcus pneumoniae* and *Staphylococcus aureus* as gram positive and *Pseudomonas aeruginosa* and *Escherichia Coli* as gram negative bacteria and their antifungal activity were studied against *Candida albicans* and *Aspergillus niger*, and ampicillin (AMP) (at 100 ng·mL^−1^) was used as a standard drug (positive control). The obtained results indicated that compound **18**, at 100 ng·mL^−1^ (Table 2) has an inhibition zone (in mm) of *S. pneumoniae* and *S. aureus* proliferation (30 and 24 mm, respectively) larger than the standard drug (20 and 22 mm) [76].

## 5. Bioactivity of 16*E*-arylidenoestrone and 16*E*-arylidenoestradiol Derivatives 

It is well known that estrogenic hormones have an important contribution to estrogen-dependent diseases, being breast cancers primarily initiated and stimulated by estrogens the majority of these conditions [77]. Consequently, the structural modification of estrone and estradiol in different positions to prepare bioactive compounds in this context has been the focus of intensive research. In this context, 16*E*-arylidenoestrone derivatives have also been prepared, and their bioactivity has been evaluated. For example, these arylidenes were studied as 17β-HSD type 1 inhibitors. This enzyme is responsible for the reduction of the 17-ketone group of estrone to afford 17β-estradiol, the most potent estrogen. Therefore, 17β-HSD type 1 is considered a relevant therapeutic target in the treatment of estrogen-dependent cancers and endometriosis [78,79,80,81]. Thus, over the years, potent 17β-HSD type 1 inhibitors have been designed and synthesized. In addition, selectivity for 17β-HSD type 1 over other isoforms and absence of estrogenic effects are of high relevance [82]. In this context, Allan et al. and Poirier et al. designed and prepared 16*E*-arylidenoestrone derivatives as 17β-HSD type 1 inhibitors, and promising results were achieved (Table 3). For example, steroid **19** (Table 3), prepared by Poirier and coworkers, presented a high inhibitory effect with an IC_50_ of 3.4 μM, which is a value lower than the observed for the positive control, estradiol (7.3 μM) [35]. In addition, Allan et al. modified estrone in different positions, namely C-6, C-16, and C-17, and the prepared structures were tested against 17β-HSD type 1 and type 2. Among all these derivatives, steroid **20** (Table 3), bearing a 4-dimethylaminobenzylidene group at C-16, presented very relevant results. In fact, at 10 μM, this derivative led to a 72% inhibition of 17β-HSD type 1 activity, while the 17β-HSD type 2 inhibition was only 13%. This research group also verified that further modifications of steroid **20** led to a loss of selectivity [83]. More recently, Wang et al. developed a new series of 16*E*-benzylidene modified 2-methoxyestradiol analogues. 2-Methoxyestradiol (2ME) is an endogenous estradiol metabolite that has been studied as an antitumor agent and several mechanisms of action for this compound have been explored. Interestingly, this steroid seems to mainly lead to neoangiogenesis inhibition, microtubule disruption, and upregulation of the extrinsic and intrinsic apoptotic pathways [84]. In this scope, Wang and coworkers used 2ME as starting material for the synthesis of 16*E*-benzylidenestradiol derivatives and their potential antitumoral interest was evaluated. Of these prepared compounds, steroid **21** (Table 3) showed a potent antiangiogenic activity. Moreover, further studies suggested that this compound suppresses the tumor growth in about 50% in human breast cancer (MCF-7) xenograft models without relevant side effects. The action mechanism studies suggested that steroid **21** targeted the epithelial to mesenchymal transition process in MCF-7 cells and inhibited human umbilical vein endothelial cells (HUVEC) migration, contributing to angiogenesis interruption [85]. 

Minu and coworkers also synthesized novel 16*E*-arylidene derivatives from estrone, and their biological activity was assessed. These compounds have been studied at NCI for their antineoplastic activity against a 60-cell lines panel and have also been tested for their in vitro estrogenic and anti-estrogenic activities, but the results obtained were not as promising as observed in other previously reported studies. Moreover, the measured ER binding affinity was also assessed, and the values determined were low [86]. In addition, novel 21*E*-benzylidene steroidal derivatives were synthesized from progesterone by Fan et al. These novel compounds were tested through MTT assay for their cytotoxicity against brine shrimp and murine Lewis lung carcinoma (LLC) cells. In general, this series of new steroids showed a weak cytotoxicity toward these cells [87]. Shan and coworkers also reported the preparation of 3β,7α,11α-trihydroxy-pregn-21-benzyliden-5-en-20-one derivatives and their cytotoxic activity. However, all these compounds showed a lower cytotoxic activity against EC109 cells than the positive control, oridonin [88]. More recently, Canário et al. prepared Δ^9,11^-estrones as potential antiproliferative agents, including a 16*E*-benzylidene derivative from Δ^9,11^-estrone. The effect of this compound on cell proliferation was assessed against several cell lines and it was verified that the introduction of a ∆^9,11^ double bond and a 16*E*-benzylidene group in estrone lead to an increase of the cytotoxic activity against the hormone-dependent breast cancer cells MCF-7 and T47-D, with IC_50_ values of 25.14 and 25.06 μM, respectively. However, when compared to 5-FU, as the positive control, this compound showed to be less potent (IC_50_ = 1.71 and 0.54 μM, respectively) [89].

## 6. Importance of Steroidal Arylidene Derivatives as Synthetic Intermediates of Bioactive Molecules

In addition to their biological activity, steroidal arylidenes are also versatile synthetic intermediates in the preparation of other bioactive structures. In fact, these steroids have been used in the introduction of diverse chemical groups present in bioactive compounds, such as oximes, hydroxyl and hydrazones [69,90,91,92,93], and are particularly useful in several heterocyclization reactions. In this context, over the years, a large number of bioactive heterocyclic steroidal derivatives have been synthesized, and some of them are already being clinically used [94,95]. Interestingly, diverse heterocyclic compounds, including arylpyrazolines and pyrazoles, arylpyrimidines, oxindoles, pyridones and pyridines as well as spiro-pyrrolidines were prepared from arylidenesteroids [4,18,42,49,53,56,76,93,96,97,98,99,100,101,102,103,104,105,106].

**Figure 5:**
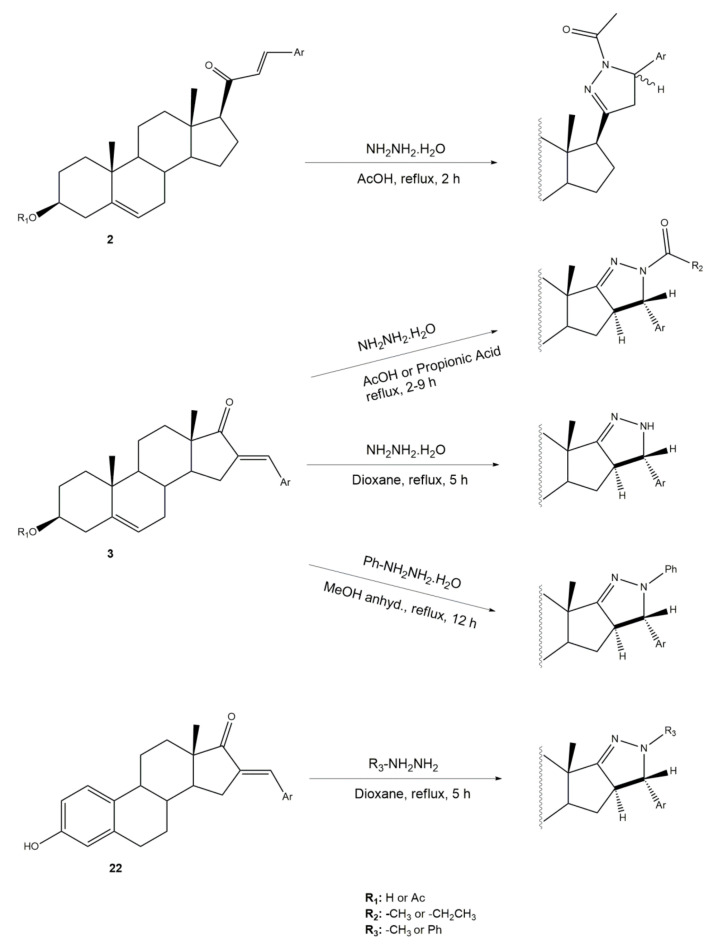
Synthesis of steroidal arylpyrazolines from 16*E*- and 21*E*-arylidenesteroids.

Pyrazolines seem to be the most frequently prepared heterocycles from steroidal arylidenes (Figure 5). A common approach to synthesize C-17 pyrazolinyl derivatives is the heterocyclization of 21*E*-arylpregnenolones in the presence of hydrazine hydrate (NH_2_·NH_2_·H_2_O) in glacial acetic acid (AcOH) under reflux during approximately 2 h [4,96,107]. Diverse approaches to obtain 16,17-pyrazolinyl steroids from 16*E*-arylidenedehydroepiandrosterone derivatives have also been reported (Figure 5). Thus, different androstane-*N*-acylated pyrazolines were prepared in the presence of NH_2_NH_2_.H_2_O in AcOH or propionic acid at reflux temperature after 2–9 h [97,100,108]. In addition, Singh et al. reported the synthesis of pyrazolinyl androstanes by using only NH_2_NH_2_.H_2_O in 1,4-dioxane under reflux for 5 h [97]. Moreover, the reaction of these 16*E*-arylideneandrostanes with phenylhydrazine hydrate in anhydrous MeOH under reflux during 12 h afforded the corresponding phenylpyrazolinyl substituted derivatives [97]. Furthermore, Naggar et al. synthesized estrone-*N*-substituted pyrazolines from 16*E*-arylidenoestrones by condensation with methylhydrazine or phenylhydrazine in refluxing dioxane for 5 h [99]. To stress that the information about the stereochemistry of 16,17-pyrazolinyl steroids is not always clear. In fact, some authors indicated that C-C bound between C-16 and C-5′ is β [97,100]. On the other hand, the study reported by El-Naggar et al. do not present any information about the stereochemistry of these compounds [99]. 

**Figure 6:**
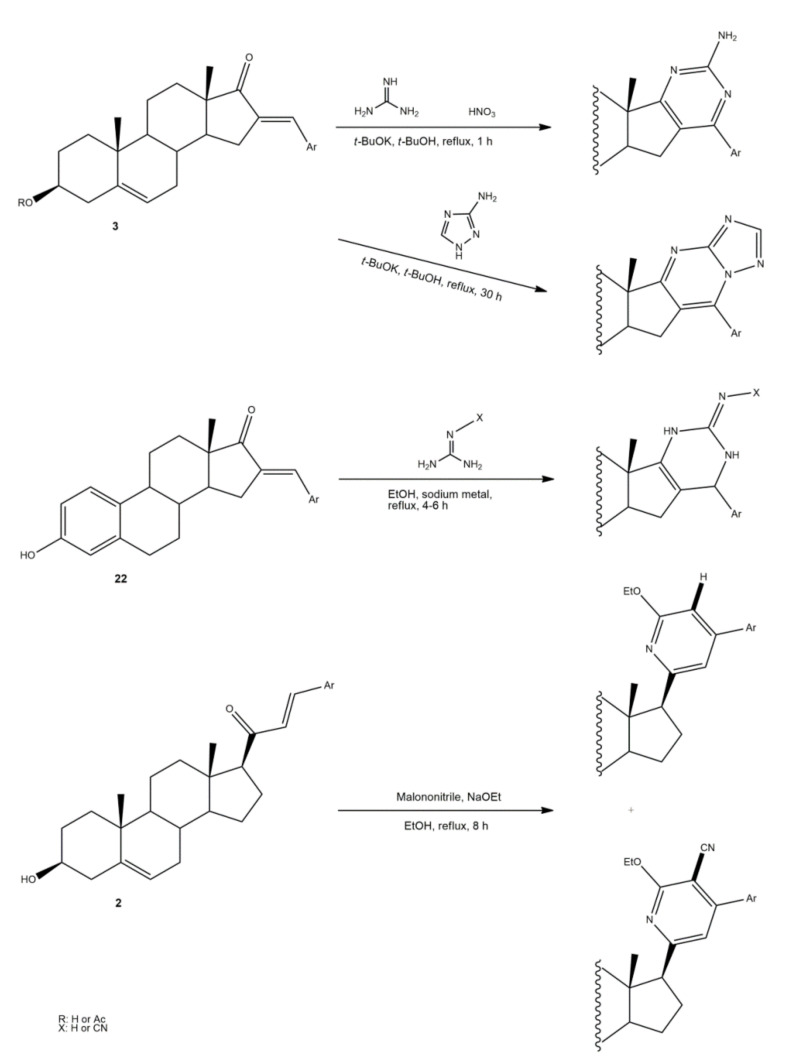
Synthesis of steroidal arylpyrimidines and pyridines from 16*E*- and 21*E*-arylidenesteroids.

The synthesis of steroidal arylpyrimidines and pyridines from 16*E-* and 21*E*-arylidenesteroids was also described (Figure 6). In fact, Ke et al. described the preparation of steroidal[17,16-*d*]pyrimidines using **3** as starting material and guanidine nitrate under basic catalysis and reflux during 1 h [49]. In addition, Huang et al. reported the synthesis of steroidal[17,16-*d*]triazolopyrimidines using 3-aminotriazole, again using a basic catalyst at reflux during 30 h [41]. Furthermore, 2’-aminopyrimidines and 2’-cyanoiminopyrimidines were prepared from 16*E*-arylidenoestrone derivatives using guanidine hydrochloride or 2-cyanoguanidine and sodium ethoxide in EtOH, and the mixture was refluxed for 4–6 h [102]. Different steroidal pyridines were also synthesized from 21*E*-arylidenesteroids in the presence of malononitrile and sodium ethoxide, at reflux temperature for 8 h, in a single reaction. Interestingly, sodium ethoxide acted as a bifunctional species, being simultaneously the ethoxy source and the promoter of reactions between α,β-unsaturated ketones and malononitrile [101].

**Figure 7:**
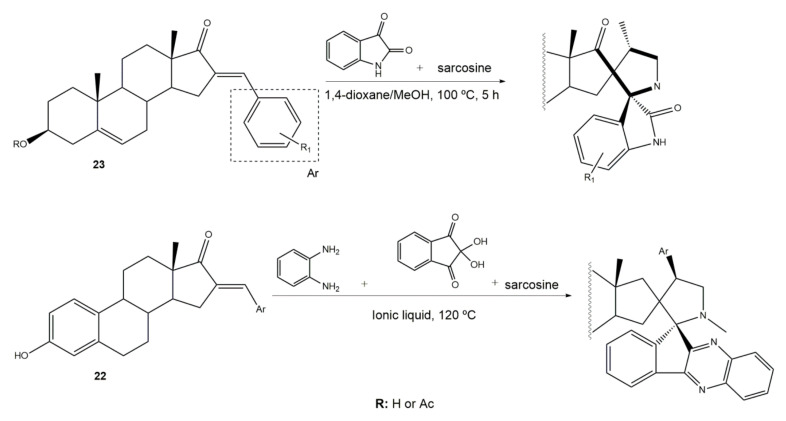
Preparation of steroidal spiro-oxindoles and spiro-pyrrolidines from 16*E*-arylidenesteroids [56,104].

The preparation of steroidal spiro-oxindoles and spiro-pyrrolidines from arylidenesteroids was reported by Yu et al. and Gavaskar et al. (Figure 7). In this context, a series of novel steroidal spiro-pyrrolidinyl oxindoles was synthesized through a 1,3-dipolar cycloaddition of azomethine ylides generated from the decarboxylative condensation of isatin and sarcosine. For this, the arylidenesteroids **23** were mixed with isatin and sarcosine in 1,4-dioxane/MeOH (1:1) and this reactional mixture was left under reflux for 5 h to afford the referred products in excellent yields [56]. More recently, Gavaskar et al. reported a 1,3-dipolar cycloaddition to prepare novel steroidal dispiropyrrolidine heterocycles. This reaction occurred through an azomethine ylide generated from 1,2-phenylenediamine, ninhydrin, sarcosine, and a 16*E*-arylidenoestrone derivative in the ionic liquid *N*-(1-acryloyl)-*N*-(4-cyclopentyl)piperazinium phosphate, previously synthesized by this group, 120 °C [104,109]. Most of these final heterocyclic products have been developed and evaluated as antiproliferative and anti-inflammatory agents, as well as aromatase and 5α-reductase (5AR) inhibitors. 5AR is the enzyme responsible for the NADPH-dependent conversion of testosterone (T) to the more potent androgen dihydrotestosterone (DHT). There are two distinct isozymes, differentially expressed in human tissues, the type 1 5AR (5AR-1) and type 2 5AR (5AR-2) [107,108]. Additionally, DHT have an important role in benign prostatic hyperplasia (BHP), a common disease in aging men [110]. These facts have contributed to the increase of the interest in finding potent 5AR inhibitors. Finasteride and dutasteride are the most effective steroidal analogs clinically used. However, both molecules cause several side effects such as erectile dysfunction, abnormal ejaculation, impotence, abnormal sexual function, decreased sexual desire, and gynecomastia [111,112]. Hence, it becomes important to develop more potent and safer 5AR inhibitors.

The most promising steroidal heterocyclic derivatives prepared from arylidenesteroids that have been reported until the moment are presented in Table 4. Of these, steroid **24** has relevant antiproliferative effects, as a very low IC_50_ values (0.24 and 0.25 μM, respectively) against HT-29 and HCT-15 cells were determined [4]. Furthermore, pyrimidine **25** has also exhibited interesting antiproliferative effects against HepG2, Huh-7 and SGC-790 cells, presenting IC_50_ values lower than positive control (5.41, 5.65 and 10.64 μM, respectively). In addition, the steroids **26**–**30** were evaluated as potential 5AR inhibitors, and some interesting results were obtained (Table 4). Compound **26** presented relevant 5AR-1 inhibitory effects in comparison with finasteride (IC_50_ values are 14.5 μM and 21.6 μM, respectively), whereas derivatives **27**–**30** revealed potent 5AR-2 inhibitory effects [96,98]. Of these, pyrazolines **29** and **30** showed the most promising results (the IC_50_ values for 5AR-2 inhibition were 7.3 and 8.2 nM, respectively), while **27** and **28** presented apparently lower inhibitory activity for 5AR-2 (the determined IC_50_ were 13.90 and 14.20 μM, respectively) [96,98]. Steroid **31** (Table 4) also showed to be a potential antiproliferative agent against NCI-H460 and HeLa cells, presenting good IC_50_ values (10.30 and 12.50 μM, respectively). Particularly, in HeLa cell line, this compound led to cell cycle arrest at S phase [18]. Yu and coworkers also reported some molecules with interesting results for antiproliferative activity, namely the steroidal spiropyrrolidinyl oxindoles **32**–**34**, synthesized from DHEA (Table 4). Particularly, pyrazolidine **32** showed good antiproliferative activity against SMMC-7721 (IC_50_ = 4.30 μM) and MCF-7 (IC_50_ = 2.06 μM) cells, being more potent than positive control (5-FU) [56]. Steroid **33** also presented relevant results in SMMC-7721 and MGC-803 cell lines (the determined IC_50_ were 6.05 and 5.79 μM, respectively). In addition, flow cytometry studies demonstrated that this compound caused cellular early apoptosis and cell cycle arrest at G2/M phase in a concentration and time-independent mode [56]. Additionally, compound **34** showed to be the most potent derivative in SMMC-7721 cells (IC_50_ = 0.71 μM) [56]. Steroid **35** (Table 4) also stood out from novel pregnenolone derivatives synthesized by Choudhary et al., presenting an interesting effect against MDA-MB 231 cells (the determined IC_50_ value for antiproliferative activity was 0.91 μM). In addition, this result showed that steroid **35** is more potent than positive control, DOX (IC_50_ = 1.23 μM) [93].

Mitchell and coworkers synthesized 16-substituted 4-azasteroids as tissue-selective androgen receptor modulators, using 16*E*-arylideneandrostane derivatives as synthetic intermediates. In general, this class of steroids displayed potent AR binding and agonist activity. Of these novel molecules, **36** (Table 4) showed the most promising results, exhibiting an osteoanabolic and tissue-selective profile in ovariectomized (OVX) and orchiectomized (ORX) rat models. In OVX rats (models with bone loss that simulate estrogen deficiency), the main evaluated parameter was bone formation rate (BFR) and it was reported as a percentage of the DHT effect at a standard dose. On the other hand, the selectivity was assessed by measuring the effects on ventral prostate (VP) and seminal vesicles (SV) through ORX male rat models. Interestingly, compound **36** exhibited a BFR of 120%, relative to DHT, an increase in the VP of 3% and a 21% of increase in SV weight, confirming its tissue selectivity (Table 4) [113]. Furthermore, the preparation and biological evaluation of a new series of 16*E*-arylidene substituted steroidal oximes was also reported from 16*E*-arylideneandrostanes [57,114]. In this context, Chattopadhaya et al. used the hollow fiber assay to assess the effects of novel 16*E*-arylideneandrostane oxime derivatives [57]. Interestingly, the oxime **37** presented the best result, exhibiting a good score for inhibition of tumoral cells in the S.C. fiber implants (4/20) and for I.P. fiber implants (6/20) (Table 4). Moreover, Dubey and coworkers also synthesized 16*E*-benzylidene substituted steroidal oximes from 16*E*-arylideneepiandrosterone derivatives [114]. These compounds were evaluated for in vitro antineoplastic activity at NCI against three cell lines. Within these oximes, the 3β-hydroxy derivative **38** (Table 4) was found as the most potent. The results indicated that in the 3 cell lines tested, NCI-H460 (lung), MCF-7 (breast) and SF-268 (central nervous system), this oxime **38** showed negative values for the percentage of cell growth (−44, −44 and −79, respectively) (Table 4) [114]. In addition, the same authors also reported the preparation of 16*E*-benzylidene substituted 3,17-dioximinoandrostene derivatives as anticancer agents and interesting results were obtained [92]. These steroids were also biologically tested at NCI against the same 3 cell lines. Of these, compound **39** showed to be the most active dioxime against the tumoral cells, presenting the lowest percentage values for cell growth in NCI-H460, MCF-7 and SF-268 cells (−11, 5, and −8, respectively) (Table 4).

## 7. Conclusions

Steroids constitute an important group of structurally related natural, semi-synthetic, and synthetic compounds with remarkable functions, including regulatory and signaling activities. In the last three decades, steroidal arylidene derivatives have been prepared and screened for a range of biological activities and used as synthetic intermediates, with special attention to bioactive heterocyclic steroids.

Arylidenesteroids are usually prepared by an aldol condensation under mild reactional conditions and mainly comprise three distinct groups based on starting materials: 2- and 16*E*-arylideneandrostane, 21*E*-arylidenepregnane, and 16*E*-arylidenoestrone derivatives. A substantial number of these steroids have shown significant antiproliferative activity when compared to reference compounds. In addition to this cytotoxic activity, a considerable number of 16*E*-arylideneandrostane derivatives were also studied as aromatase inhibitors with promising results, and a few 16*E*-arylidenoestrone derivatives were studied as 17β-HSD type 1 inhibitors. In general, the most active arylidenesteroids usually comprise heteroatoms or halogens on their structures.

In addition, steroidal arylidene derivatives have also emerged as very important synthetic intermediates to obtain steroidal heterocycles as arylpyrazolines and pyrazoles, arylpyrimidines and pyridines, spiro-oxindoles, and spiro-pyrrolidines, among other bioactive systems. Within these, arylpyrazoline derivatives stand out as 5AR inhibitors and antiproliferative agents.

In conclusion, due to the straightforward synthesis of arylidenesteroids and their bioactivity, as well as the inherent chemical reactivity of α,β-unsaturated ketones, useful in the preparation of other derivatives, this class of compounds has been of high interest in the last years. This review highlighted the most relevant points in this context and evidenced, for the first time, the great relevance of this particular group of steroids, which should be of high interest in further studies looking to new, more active, and safer bioactive compounds with potential clinical interest.

## Figures and Tables

**Table 1:** Examples of relevant bioactive 2- and 16*E*-arylideneandrostane derivatives. Positive controls, when shown, are identified by (+) symbol.

Compound	Bioactivity Data						Ref.
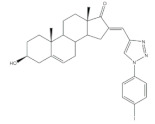 2	Antiproliferative activity (IC50 μM)						[16]
	Cell line	2		5-FU (+)			
	HepG2	9.10		10.59			
	MCF-7	9.18		28.11			
	Cell cycle arrest at G2 phase in HepG2						
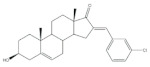 3	Antiproliferative activity (IC_50_ ± SEM μM)						[60]
	Cell line	3		Etoposide (+)			
	KB	0.6 ± 2.0		2.8 ± 16.8			
	T47-D	1.7 ± 14.8		1.2 ± 8			
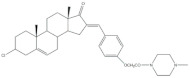 4	Antiproliferative activity (IC_50_ μM)						[61]
	Cell line		4				
	CCRF-CEM		3.94				
	K-562		2.61				
	RPMI-8226		6.90				
	SR		1.79				
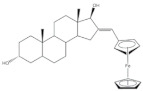 5	Antiproliferative activity (IC_50_ μM ± SD μM)						[59]
	Cell line		5		Cis (+)		
	HT-29		1.2 ± 0.4		66 ± 2		
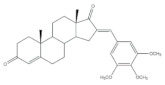 6	Cytotoxic activity–in vivo hollow fiber assay (score)						[57]
		6				Taxol (+)	
	I.P.	2				Data not shown	
	S.C.	8					
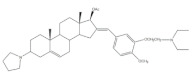 7	Cytotoxic activity–in vivo hollow fiber assay (score)						[62]
		7				Taxol (+)	
	I.P.	12				Data not shown	
	S.C.	8					
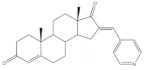 8	Aromatase inhibitory activity (IC_50_ μM)						[70]
	8		Aminoglutethimide (+)				
	5.2		28.5				
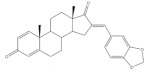 9	Aromatase inhibitory activity (IC_50_ μM)						[70]
	9		Aminoglutethimide (+)				
	6.4		28.5				
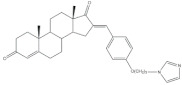 10	Aromatase inhibitory activity (IC_50_ μM)						[71]
	10		Aminoglutethimide (+)				
	4.4		28.5				
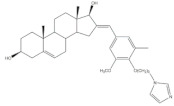 11	Aromatase inhibitory activity (IC_50_ μM)						[71]
	11		Aminoglutethimide (+)				
	2.4		28.5				
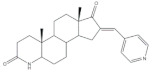 12	Anti-inflammatory activity TNF-α levels (pg.mg^−1^protein ± SD)						[72]
	12	CEL (+)				DEX (+)	
	88.6 ± 1.8	68.2 ± 1.1				89.6 ± 2.0	
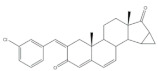 13	Anti-inflammatory activity (IC_50_ μM) (NO release of LPS-activated mouse microglial cell line BV2)						[73]
	13		Minocycline (+)				
	2.69		5.97				
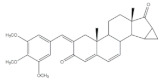 14	Anti-inflammatory activity (IC_50_ μM) (NO release of LPS-activated mouse microglial cell line BV2)						[73]
	14		Minocycline (+)				
	3.28		5.97				

**Table 2:** The most active 21*E*-arylidenepregnene derivatives. Positive controls, when shown, are identified by (+) symbol**.**

Compound	Bioactivity Data			Ref.
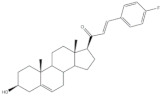 15	Antiproliferative activity (IC_50_ μM)			[69]
	Cell line	15		
	HCT-15	0.81		
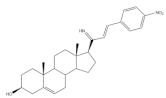 16	Antiproliferative activity (IC_50_ μM)			[69]
	Cell line	16		
	MCF-7	0.60		
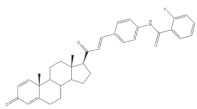 17	Antiproliferative activity Growth inhibition (%)			[38]
	Cell line	17	Cis (+)	
	HeLa	58	99	
	MCF-7	64	88	
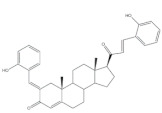 18	Antimicrobial activity Zone of inhibition (mm)			[76]
	Gram positive	18	AMP (+)	
	*Streptococcus pneumoniae*	30	20	
	*Staphylococcus aureus*	24	22	

**Table 3:** The most active 16*E*-arylidenoestrone and -estradiol derivatives. Positive controls, when shown, are identified by (+) symbol.

Compound	Bioactivity Data		Ref.
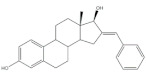 19	17β-HSD1 inhibitory activity (IC_50_ μM)		[35]
	19	Estradiol (+)	
	3.4	7.3	
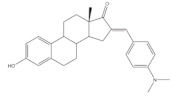 20	17β-HSD inhibitory activity (% at 10 μM)		[83]
	20		
	Type 1	Type 2	
	72	13	
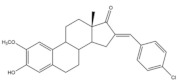 21	Tumor weight reduction (%) Human breast cancer (MCF-7) xenografts models		[85]
	21 (45 mg/kg/day)	2ME (+) (45 mg/kg/day)	
	About 50% Significative reduction compared with negative control	About 50% Significative reduction compared with negative control	

**Table 4:** The most active heterocyclic steroidal derivatives obtained from arylidenesteroids. Positive controls, when shown, are identified by (+) symbol.

Compound	Bioactivity Data				Ref.
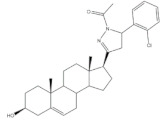 24	Antiproliferative activity (IC_50_ μM)				[4]
	Cell line	24			
	HT-29	0.24			
	HCT-15	0.25			
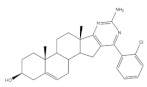 25	Antiproliferative activity (IC_50_ μM)				[49]
	Cell line	25	5-FU (+)		
	HepG-2	5.41	>100		
	Huh-7	5.65	>95		
	SGC-790	10.64	>100		
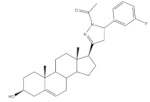 26	5AR-1 Inhibition (IC_50_ ± SEM μM)				[96]
	26	Finasteride (+)			
	14.50 ± 0.48	21.6 ± 0.62			
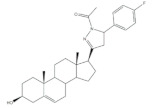 27	5AR-2 Inhibition (IC_50_ ± SEM μM)				[96]
	27	Finasteride (+)			
	13.90 ± 0.75	15.4 ± 0.58			
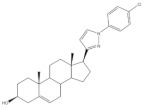 28	5AR-2 Inhibition (IC_50_ ± SEM μM)				[96]
	28	Finasteride (+)			
	14.20 ± 0.75	15.4 ± 0.58			
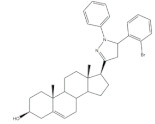 29	5AR-2 Inhibition (IC_50_ ± SEM nM)				[98]
	29	Finasteride (+)			
	7.30 ± 0.62	2.4 ± 0.15			
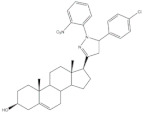 30	5AR-2 Inhibition (IC_50_ ± SEM nM)				[98]
	30	Finasteride (+)			
	8.20 ± 0.55	2.4 ± 0.58			
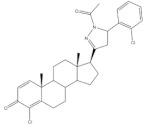 31	Antiproliferative activity (IC_50_ μg.mL^−1^)				[18]
	Cell line	31	5-FU (+)	Cis (+)	
	NCI-H460	10.30	2.48	0.699	
	HeLa	12.50	0.887	2.03	
	Cell cycle arrest at S phase in HeLa cells				
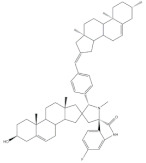 32	Antiproliferative activity (IC_50_ μM)				[56]
	Cell line	32	5-FU (+)		
	SMMC-7721	4.30	9.78		
	MCF-7	2.06	7.54		
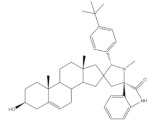 33	Antiproliferative activity (IC_50_ ± SEM μM)				[56]
	Cell line	33	5-FU (+)		
	SMMC-7721	6.05 ± 0.48	9.78 ± 0.99		
	MGC-803	5.79 ± 0.76	6.92 ± 0.35		
	Cell cycle arrest at G2/M phase in MGC cells				
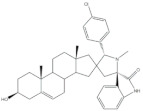 34	Antiproliferative activity (IC_50_ ± SEM μM)				[56]
	Cell line	34	5-FU (+)		
	SMMC-7721	0.71 ± 0.11	9.78 ± 0.99		
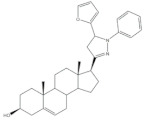 35	Antiproliferative activity (IC_50_ μM)				[93]
	Cell line	35	DOX (+)		
	MDA-MB 231	0.91	1.23		
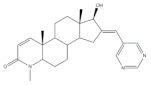 36	Osteoanabolic activity and tissue-selectivity (% of the effect of DHT)				[113]
	OVX	36	DHT		
	BRF	120	100		
	ORX				
	VP	3	100		
	SV	21	100		
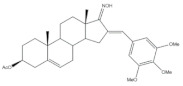 37	Cytotoxic activity-in vivo hollow fiber assay (score)				[57]
		37	Taxol (+)		
	I.P.	4	Data not shown		
	S.C.	6			
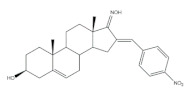 38	Antiproliferative activity Cell growth (%)				[114]
	Cell line	38			
	NCI-H460	−44			
	MFC-7	−44			
	SF-268	−79			
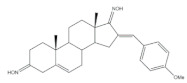 39	Antiproliferative activity Cell growth (%)				[92]
	Cell line	39			
	NCI-H460	−11			
	MCF-7	5			
	SF-268	−8			
